# Molecular Characterization of Membrane Steroid Receptors in Hormone-Sensitive Cancers

**DOI:** 10.3390/cells10112999

**Published:** 2021-11-03

**Authors:** Mirco Masi, Marco Racchi, Cristina Travelli, Emanuela Corsini, Erica Buoso

**Affiliations:** 1 Dipartimento di Scienze del Farmaco, Università Degli Studi di Pavia, Viale Taramelli 12/14, 27100 Pavia, Italy; mirco.masi@iusspavia.it (M.M.); racchi@unipv.it (M.R.); cristina.travelli@unipv.it (C.T.); 2 Scuola Universitaria Superiore IUSS, Piazza della Vittoria 15, 27100 Pavia, Italy; 3 Laboratory of Toxicology, Dipartimento di Scienze Farmacologiche e Biomolecolari, Università Degli Studi di Milano, Via Balzaretti 9, 20133 Milano, Italy

**Keywords:** ZIP9, OXER1, GPRC6A, TRPM8, GPER, mPR, PGRMC, breast cancer, prostate cancer, ovarian cancer, endometrial cancer

## Abstract

Cancer is one of the most common causes of death worldwide, and its development is a result of the complex interaction of genetic factors, environmental cues, and aging. Hormone-sensitive cancers depend on the action of one or more hormones for their development and progression. Sex steroids and corticosteroids can regulate different physiological functions, including metabolism, growth, and proliferation, through their interaction with specific nuclear receptors, that can transcriptionally regulate target genes via their genomic actions. Therefore, interference with hormones’ activities, e.g., deregulation of their production and downstream pathways or the exposition to exogenous hormone-active substances such as endocrine-disrupting chemicals (EDCs), can affect the regulation of their correlated pathways and trigger the neoplastic transformation. Although nuclear receptors account for most hormone-related biologic effects and their slow genomic responses are well-studied, less-known membrane receptors are emerging for their ability to mediate steroid hormones effects through the activation of rapid non-genomic responses also involved in the development of hormone-sensitive cancers. This review aims to collect pre-clinical and clinical data on these extranuclear receptors not only to draw attention to their emerging role in cancer development and progression but also to highlight their dual role as tumor microenvironment players and potential candidate drug targets.

## 1. Introduction

Cancer is one of the leading causes of death worldwide, with nearly 10 million deaths reported in 2020 [1]. The transformation of normal cells in tumor cells is a multi-step process—from pre-cancerous lesions to the malignant tumor—that results from the interaction between genetic factors; environmental factors, such as chemical, physical, and biological carcinogens (as external agents); and aging. Among all types of tumors, those types that strictly depend on one or more hormones for growth, spread, and survival are defined hormone-sensitive cancers or hormone-dependent cancers. These include breast, ovarian, uterine (or endometrial), prostate, testis, and thyroid cancer [2].

Estrogen and progesterone appear to be the main sex hormones involved in growth of breast and uterine cancers, while ovarian and prostate cancers are mainly related to estrogens and androgens, respectively [2]. Breast and prostate cancers alone accounted for 2.26 and 1.41 million cases, respectively, in 2020. Although the type of tumor-initiating cues for hormone-sensitive cancers can be wide-ranging, the promotion event and subsequent growth and proliferation are driven by a hormone [2]. In addition to endogenous hormones, exogenous hormone-active substances, i.e., endocrine-active substances (EASs) and endocrine-disrupting chemicals (EDCs), can interact or interfere with the normal hormonal action and drive cell proliferation, thus increasing the cell division rate and, therefore, the consequent number of random genetic errors capable of triggering the cancerous transformation [3,4].

Steroid hormones that are derived from cholesterol are divided into sex steroids (estrogens, androgens, and progestogens) and corticosteroids (glucocorticoids and mineralocorticoids). These hormones, by interacting with specific nuclear Steroid Receptors (nSRs), can regulate different physiological functions, including growth, development, metabolism, and reproduction [5]. Therefore, their abnormal production, deregulation of their signaling pathways, and interference with their action all play a role in supporting the growth, proliferation, and spread of hormone-sensitive cancers. Indeed, treatments for these cancer types are mostly based on the interruption of the hormonal stimuli, e.g., with inhibitors for key enzymes involved in hormone synthesis and with nSR antagonists. In addition to nSRs that produce slow genomic responses, other extranuclear steroid receptors have also been reported to mediate the biological effects of steroids. These membrane steroid receptors (mSRs) are cell surface receptors capable of mediating rapid and non-genomic signaling by modulating different intracellular molecular pathways that, through distinct signaling cascades, contribute to the regulation of steroid hormone-correlated functions [6,7].

The aim of this review is to gather the existing scientific knowledge on mSRs to show their pivotal role in mediating the effects of steroid hormones and hormone-active substances in both physiologic and pathologic contexts; we also seek to elucidate their role as key players in shaping the cancer environment and, therefore, as potential drug targets.

## 2. Membrane Steroid Receptors and Their Role in Hormone-Sensitive Cancers

Steroid hormones can regulate gene expression in the nucleus but can also exert non-genomic functions by binding at or near the plasma membrane to induce rapid changes in cell physiology. Genetic and pharmacologic studies demonstrate mSRs function in different cell types and tissues where they respond to steroid ligands independently of the corresponding nSR. Some nSRs can exert their function at the plasma membrane, e.g., through post-translational modifications via palmitoylation or association with membrane scaffold proteins, to trigger cellular responses independently of their function as transcription factors [8]. In contrast, mSRs are integral membrane steroid receptors that feature transmembrane protein domains and, in response to steroid hormone binding, can activate or inactivate other proteins and second messenger cascades. Here, we focus on mSRs—which are lesser known and studied compared to their corresponding nSR—to dissect their molecular function and their pharmacology in hormone-sensitive cancers.

### 2.1. Membrane Androgen Receptors

In addition to the nSR known as Androgen Receptor (AR), other molecular mediators of the androgenic action include different plasma membrane receptors, namely, the Zinc Transporter Member 9 (ZIP9), the Oxoeicosanoid Receptor 1 (OXER1), the G protein-coupled receptor family C group 6 member A (GPRC6A), the Ca^2+^ channel Transient Receptor Potential Cation Channel Subfamily M (Melastatin) Member 8 (TRPM8) [9], and the L-type Voltage-dependent Calcium Channel (CaV1.2) [10].

#### 2.1.1. ZIP9

**Figure 1:**
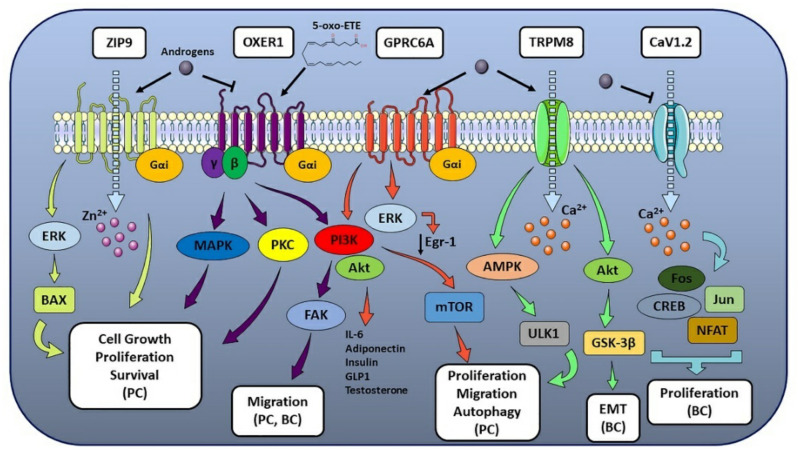
mAR-associated pathways and their effect on hormone-sensitive cancer progression. The figure illustrates the molecular pathways correlated to the different mARs and their biological effects on multiple hormone-sensitive cancer types. When one or more tumor-related processes were reported only for a specific cancer type, the latter was made explicit in the figure and put in parentheses (e.g., breast cancer as BC, prostate cancer as PC) (see text for details).

ZIP9 (also known as Zrt- and Irt-like Protein 9 and Solute Carrier family 39 member 9, SLC39A9) is a Zn^2+^ transporter protein involved in the Zn^2+^ influx from the extracellular space to the cytoplasm [11] and a membrane Androgen Receptor (mAR) coupled to G proteins [12]. ZIP9, encoded by the SLC39A9 gene in human [12], is member 9 out of 14 ZIP family proteins and is present in three isoforms with different lengths and molecular weights. Unlike other ZIP proteins that feature 8 Trans-Membrane (TM) domains and an extracellular C-terminus, ZIP9 has a 7TM structure with an intracellular C-terminal domain and is the only member of the family to signal via G protein [12]. In particular, ZIP9 is coupled to a stimulatory Gα protein (Gαs), and, upon activation, it mediates Mitogen-Activated Protein kinase (MAPK) and Zn^2+^ signaling [13]. This dual role of Zn^2+^ transporter and mAR is exerted through G proteins involved in apoptotic pathways activated by androgens [12]. Indeed, testosterone exhibits high affinity for ZIP9 and has been demonstrated to act as an agonist for this receptor, while other endogenous androgens such as androstenedione and dihydrotestosterone (DHT) display low affinity [12]. Since Zn^2+^ is required for the structure of different proteins (e.g., zinc-finger-containing transcriptional factors and Zn^2+^-dependent metalloenzymes [14]) and for several signaling pathways involved in cell growth, proliferation, and apoptosis [14], Zn^2+^ homeostasis is pivotal for human health. Therefore, its dysregulation has been associated with different pathologies, including inflammation, diabetes, and cancer [14]. In this regard, ZIP9 has been found highly expressed and bound by androgen hormones in ovarian, breast and prostate cancer cells [15], and testosterone has been demonstrated to increase intracellular Zn^2+^ levels in the same hormone-sensitive tumors, where it led to high Zn^2+^ concentration-mediated apoptosis [12,16]. ZIP9 has been shown to mediate a testosterone-induced, AR-independent increase of cell migration in metastatic prostate cancer cells [17]. In contrast to previous literature data on ZIP9, testosterone treatment in human breast and prostate cancer cell lines results in the activation of an inhibitory Gα protein (Gαi) [18]. Interestingly, ZIP9 was found significantly up-regulated in breast cancer tissues compared with normal breast tissues [19], the ZIP9-encoding SLC39A9 gene has been observed to form a fusion transcript with MAP3K9 gene (encoding for Mitogen-Activated Protein Kinase 9, MAP3K9), and the resulting fusion gene has been reported with repetitive incidence in different types of breast cancer [20] (Figure 1).

#### 2.1.2. OXER1

OXER1 (previously known as G Protein-coupled Receptor 170 (GPR170), hGPCR48, HGPCR48, TG1019 or R527) is a G protein-coupled receptor (GPCR) coupled to a Gαi [21] and activated by 5-oxoeicosatretraenoic acid (5-oxo-ETE), 5-lipoxygenase (5-LOX) and peroxidase metabolite of arachidonic acid [22]. OXER1 mediates several intracellular actions, including steroidogenesis stimulation, immune and inflammatory responses, cell proliferation and survival [23]. Upon 5-oxo-ETE binding, OXER1 activation results in the Gαi-mediated inhibition of cAMP production and Gβγ-mediated induction of Ca^2+^ mobilization, although Gβγ is responsible for mediating several other signaling pathways [22,24]. These include chemotactic response and actin polymerization in different leukocyte types (e.g., neutrophils, eosinophils, and monocytes) [24,25], activation of PI3K and Akt (also known as Protein Kinase B, PKB), focal adhesion kinase (FAK), extracellular signal-regulated kinases 1/2 (ERK1/2), Phospholipase A2 (PLA2) and modulation of different Protein Kinase C (PKC) isoforms [26,27]. Most of these pathways are involved, even in different cellular contexts, in proinflammatory responses, infiltration and cell migration [9]. Despite the fact that most literature data are focused on OXER1 role in immune context, their role in the progression of different hormone-related cancers has also been investigated. Literature data confirmed that OXER1 is highly expressed in cancer cells and tissues, including prostate and breast [28,29], where it has been observed to mediate survival-promoting effects and inhibition of cell apoptosis, when activated by its endogenous ligand 5-oxo-ETE [23,29]. Cancer cells display high levels of 5-hydroxy-eicosatetraenoic acid dehydrogenase (5-HEDH) that can convert inflammatory cell-derived 5-hydroxyeicosatetraenoic acid (5S-HETE) to 5-oxo-ETE, especially when the cells are stressed [30]. 5-oxo-ETE produced within the tumor microenvironment (TME) can support OXER1-mediated cancer cell growth and promote further infiltration of inflammatory cells. Both prostate cancer-derived cell lines and prostate cancer tissue from patients contain high levels of OXER1 at both mRNA and protein levels [23,29]. In addition, membrane staining for OXER1 has been found to be significantly increased in prostate cancerous tissues compared to non-cancerous ones [18], supporting OXER1’s role in prostate cancer cell growth and metastasis [23,31]. Indeed, OXER1 mediates cell proliferation, survival-promoting effects, and inhibition of cell apoptosis in prostate cancer cells, as well as adhesion, migration and invasion through its major signaling pathways involving p38α, PI3K/Akt and FAK [31]. OXER1 has also been identified as a specific mAR, and testosterone acts as an antagonist on OXER1-mediated PI3K, FAK and p38α signaling pathways, resulting in the inhibition of cell migration and metastasis [31,32] (Figure 1). OXER1 can also mediate androgen actions that antagonize the effects of 5-oxo-ETE, providing a novel link between steroid and lipid actions and an interesting target for therapeutic intervention [31]. In addition, since rapid androgen actions have been reported in classical AR-lacking breast cancer models [33] and membrane androgen binding sites have been observed in breast carcinoma cells [34], OXER1 has been recently proposed as a potential drug target in breast cancer [35]. Indeed, OXER1 role in malignant cell growth was firstly reported in MDA-MB-231 and MCF-7 breast cancer cell lines [28], which express high levels of OXER1 protein [18]. In agreement, inhibition of 5-oxo-ETE production by blocking 12-LOX and 5-LOX results in reduced proliferation and induced cytotoxicity and apoptosis, further supporting OXER1 role in breast cancer [36,37]. Data mining analysis also reveals that high OXER1 expression in tumor breast invasive carcinoma (TCGA-1097) correlates with a worse overall survival probability [38]. In addition, a recent study based on RNA-sequencing data proposed OXER1 as a key gene and possible molecular marker of tumorigenesis of Hormone-Receptor-positive/Human Epidermal Growth Factor Receptor 2 (also known as Receptor Tyrosine-Protein Kinase erbB-2, cluster of differentiation 340 (CD340), or proto-oncogene Neu)-negative (HR(+)/HER2(−)) breast cancer in adolescents and young adults [39].

#### 2.1.3. GPRC6A

GPRC6A is a Ca^2+^ and amino acid sensing GPCR (widely expressed both in humans and in rodents) whose activity has been linked to rapid, non-genomic signaling responses exerted by androgens [40]. This receptor is involved in different physiological and pathological contexts, including male fertility, insulin secretion, bone and energy metabolism, inflammatory responses, and androgen production [9]. GPRC6A is coupled to a Gαi protein and has been proposed to be a multi-sensing receptor, since its activation can be induced by Ca^2+^, Mg^2+^, osteocalcin, steroid hormones, and a variety of amino acids [40]. In this regard, GPRC6A has been demonstrated to mediate rapid and non-genomic signaling in response to androgen binding in AR-lacking cells, when overexpressed in these cellular models, while its silencing impaired exogenous androgen response [40]. As for the ability of this receptor to bind androgens, both computational analyses and in vitro assays demonstrated that testosterone can bind GPRC6A, leading to ERK1/2 phosphorylation and activation and to a decrease in tumor-suppressor Early Growth Response Protein 1 (Egr-1, also known as Zinc Finger protein 268, ZNF268, or Nerve Growth Factor-Induced protein A, NGFI-A) [40]. In addition, GPRC6A activation in different tissues led to the production of different cytokines (including IL-6 and adiponectin) and hormones (insulin, Glucagon-Like Peptide 1 (GLP-1) and testosterone) [40]. Noteworthy, compared to normal prostate cells, GPRC6A upregulation was observed in different prostate cancer cell lines, where its activation led to an increase in chemotaxis and cell proliferation in vitro [40]. Conversely, ablation of GPRC6A in prostate cancer mice xenografts resulted in decreased tumor progression and enhanced survival [41]. Indeed, testosterone binding to GPRC6A has been demonstrated to activate ERK, Akt and mammalian Target of Rapamycin (mTOR) signaling pathways in a time and dose-dependent manner, resulting in an increased cell proliferation and inhibition of autophagy in prostate cancer cells [42] (Figure 1). Noteworthy, the association between GPRC6A gene and prostate cancer risk has been widely reported in Eastern Asian populations [43,44,45], and the rs2274911 polymorphism in GPRC6A has been associated with increased risk of prostate cancer, since this mutation promotes prostate cancer cell proliferation and is associated with increased prostate-specific antigen (PSA) serum levels [46]. In addition, clinical and in vitro data revealed that GPRC6A is associated with aggressive prostate cancer, with a pivotal role in cell proliferation, migration, invasion, and Epithelial-Mesenchymal Transition (EMT) [47].

#### 2.1.4. TRPM8

TRPM8 (also known as the Cold and Menthol Receptor 1, CMR1) is an androgen-regulated, Ca^2+^-selective cation channel sensitive to cold physical stimulus, menthol and icilin (AG-3-5), located at the endoplasmic reticulum and plasma membranes of androgen-responsive cells [9]. Indeed, TRPM8 has been recognized as an androgen-related receptor due to its androgen binding affinity and steroid specificity [48], and this strong binding of androgens, such as testosterone and DHT, affects its Ca^2+^ channeling activity [49]. TRPM8 is widely expressed in different tissues, including hepatic and intestinal tissues, peripheral nervous system, male urogenital tract, and cancer tissues [50]. Indeed, TRPM8 is involved in the regulation of several key processes, including inflammatory and immunomodulatory responses, cell proliferation, migration, and apoptosis [51]. In this regard, TRPM8 is a possible prostate cancer biomarker [52,53] reported to be significantly increased in early-stage prostate cancer, while its expression is significantly decreased in advanced stages androgen-independent prostate tumors [54]. Moreover, TRPM8 is highly expressed in the prostate epithelium, its levels rise in primary and hormone naïve prostate cancer metastasis [55], and several clinical data report TRPM8 among other candidate gene markers for metastatic prostate [56,57] and TRPM8 mRNA levels were found significantly upregulated in prostate cancer samples [58] and correlated with those of kallikrein-3 (KLK3, also known as PSA, gamma-seminoprotein or P-30 antigen) [59]. Although restricted to a limited number of studies, TRPM8 involvement in tumor progression has also been investigated in breast cancer since it was found overexpressed in human Breast Ductal Adenocarcinoma (hBDA) samples [60]. In this regard, TRPM8 has been reported to activate Akt and Glycogen Synthase Kinase 3 beta (GSK-3β) pathway to promote EMT and breast cancer aggressiveness [61] and to regulate proliferation, migration, and autophagy by activating AMP-Activated Protein Kinase (AMPK) and Unc-51 Like Autophagy Activating Kinase (ULK1) pathway [62] (Figure 1). Furthermore, in vitro and clinical data support the role of TRPM8 as a biomarker for poor clinical outcome prediction in estrogen receptor (ER)-negative breast cancer patients [63,64].

#### 2.1.5. CaV1.2

CaV1.2 is a member of the L-type Voltage-Gated Ca^2+^ Channel (VGCC) family (CaV1.1–CaV1.4), known to play pivotal roles in the regulation of Ca^2+^ homeostasis, secretion, and tissue development. L-type VGCC are multi-subunit channels formed by three distinct proteins, namely, CaVα1, CaVα2δ and CaVβ [65]. The ion-conducting channel is formed by the four CaVα1 subunits, which have multiple splice variants that confer their pleiotropic effects and their association with a wide range of pathologies, including cancer [65]. Noteworthy, an increased intracellular Ca^2+^ influx induced by CaV1.2 can lead to the activation of Nuclear Factor of Activated T cells (NFAT) via calcineurin-mediated dephosphorylation and nuclear translocation [66,67]. In this regard, increased NFAT transcriptional activity has been implicated with increased cancer progression events, including proliferation, migration, invasion, autophagy, and tumor neo-angiogenesis [67,68]. Cav1.2-mediated Ca^2+^ influx has been associated with the activation of transcription factors Fos; c-Jun; cAMP Response Element-Binding protein (CREB); NFAT; and the expression of D-cyclins to control G1/S transition and, thus, cell proliferation [69,70] (Figure 1). Interestingly, not only testosterone has been demonstrated to bind and inhibit CaV1.2-induced Ca^2+^ influx [71] but also 5α-dihydrotestosterone decreased CaV1.2 expression in the luminal breast cancer MCF-7 cells, affecting their viability and proliferation [70].

### 2.2. Membrane Estrogen Receptors

nSRs, known as estrogen receptors alpha and beta (ERα and ERβ), are the most characterized and well-studied receptors known to exert the effects of endogenous and exogenous estrogens, estrogen-like substances and xenoestrogens. However, other molecular mediators of estrogenic action are located at the cellular plasma membrane and include G-Protein Estrogen Receptor (GPER), ERx, ER-X and Gq-coupled membrane Estrogen Receptor (Gq-mER) [72]. In addition, Voltage-Gated Sodium Channel Nav1.2 is also reported to bind estrogens and to activate molecular cascades upon their binding [10].

#### 2.2.1. GPER

While for ER-X and Gq-mER there are almost no literature data on their possible role in cancer progression [73], and for ERx available data are limited to in vitro observations regarding breast cancer [74], GPER (previously known as G Protein-coupled Receptor 30, GPR30) has been associated to the modulation of signaling pathways involved in tumor growth both in vitro and in vivo [75,76]. GPER is a 7TM receptor coupled to G protein (encoded by GPER gene in humans) capable of binding endogenous estradiol (E2) with high affinity and responsible for the activation of rapid, non-genomic estrogenic effects [77]. GPER is a member of the Rhodopsin-like GPCR family mainly localized at the cellular plasma membrane, where it has been shown to signal through the activation of both Gαs and Gαi [78,79]. Its activation results in the induction of Ca^2+^ mobilization and synthesis of phosphatidylinositol (3,4,5)-trisphosphate (PIP3), and several studies provided evidence of GPER localization also in intracellular compartments, including the endoplasmic reticulum, Golgi apparatus and nucleus [80]. However, its distribution appears to vary depending on species, tissue, and cell type and to dynamically change in response to specific environmental signals. GPER is expressed in different tissues, including breast, prostate, and ovary, as well as in immune cells [81,82], and its expression is suggested to be species-, gender-, tissue- and age-dependent [83]. In addition, GPER abundance has been reported to be developmentally regulated, since its expression in elongating ducts of the mammary gland not only is lower during puberty and increases during sexual maturity but also appears to be dependent on estrous cycle [78]. These findings are of pivotal interest not only in the investigation of GPER physiological function but also for its pathologic role in different contexts, including metabolic functions, reproduction, immune regulation, and cancer development and progression [82,84]. GPER expression has been associated with metastasis formation, tumor size and recurrence in both breast and ovarian cancer [85], and with the expression of a gene signature involved in the metastasis of ER-negative breast tumors [86]. In addition, GPER activation has been observed to play a critical role in breast cancer growth, proliferation, migration, and metastasis [76] via the modulation of different molecular pathways. These include the stabilization of the F-actin cytoskeleton and the upregulation of Yes-Associated Protein 1 (YAP) and Transcriptional coactivator with a PDZ-binding domain (TAZ) via the activation of Gαq-11, Phospholipase C beta (PLCβ)/PKC and Rho/Rho-associated protein kinase (ROCK) signaling [87,88]; the downregulation of microRNA-124 (miR124) and miR-148a to promote proliferation and support immune invasion via the upregulation of CD151, HOX Transcript Antisense RNA (HOTAIR) and Histocompatibility Antigen, class I, G (HLA-G) [89,90,91]; the enhancement of Fibronectin (FN) matrix assembly and anchorage-independent growth [92]; and cell survival through the activation of epidermal growth factor receptor (EGFR)/ERK/c-Fos/Activator Protein 1 (AP-1) for Sirtuin 1 (SIRT1, also known as NAD-dependent deacetylase sirtuin-1) upregulation [93], MAPK/ERK/Tripartite Motif Containing 2 (TRIM-2) for Bcl-2-like protein 11 (BIM) downregulation [94], and EGFR/PI3K for Forkhead Box O3s (FOXO3a) inhibition [95]. Moreover, GPER appears to be required for stemness maintenance in cancer stem cells via PKA/Bcl-2 associated agonist of cell death (BAD) pathway [96] and the development of chemoresistance via EGFR/ERK/Akt-mediated ATP Binding Cassette, Subfamily G, Member 2 (ABCG2) expression [97]. This is in line with the increasing evidence that GPER expression and activation is correlated with a poor response to chemotherapy with selective estrogen receptor modulators [98,99]. However, GPER role in breast cancer still needs to be fully clarified due to contrasting literature data on its significance as prognosis predictor [100,101,102]. This could be attributed not only to the variation of GPER expression during carcinogenesis due to the progressive hypermethylation of its promoter [103] but also to GPER different role depending on the specific breast cancer context.

Clinical data reported a correlation between GPER expression and poor prognosis also in other female reproductive cancers [104]. GPER has been proposed to act as tumor suppressor in ovarian cancer [105] and to have, together with Wnt pathway modulator Dickkopf 2 (Dkk2) expression, a positive prognostic impact in ovarian cancer patients [106,107]. GPER not only suppresses the proliferation of ovarian cancer cells by blocking tubulin polymerization [108,109], but its activation also led to a transcriptome response associated with growth inhibition in ovarian cancer cells [110] and triggered a ERK1/2-mediated trimethylation of Histone H3 at Lysine 4 (H3K4me3) to repress migration and proliferation in vitro [111]. However, contrasting evidence supports the role of GPER in mediating proliferation, migration, and invasion in vitro—through the expression of c-Fos, cyclin D1, Matrix Metallopeptidase 2 (MMP2, also known as gelatinase A, GELA or 72 kDa type IV collagenase) and MMP9 9 (also known as gelatinase B, GELB)—in both an E2-mediated [112,113] and a ligand-independent manner [114]. In endometrial cancer, GPER has been observed to promote cell proliferation by enhancing the expression of aromatase, nuclear hormone receptors Steroidogenic Factor 1 (SF-1) and Liver Receptor Homolog-1 (LRH-1) via a PI3K/Akt- and MAPK-mediated mechanism [115], and through a GPER/EGFR/ERK/Egr-1 transduction pathway resulting in the expression of cyclin D1 and Connective Tissue Growth Factor (CTGF, also known as CCN2) [116]. Moreover, upon E2 binding, GPER also regulates in vitro endometrial cancer cells motility and anchorage-independent growth through Diacylglycerol Kinase alpha (DGKα) [117]. GPER pro-tumorigenic role in endometrial cancer is also supported by evidence reporting anti-EMT effects by GPER targeting via miR-195 [118] and cell proliferation via miR-424 [119].

**Figure 2:**
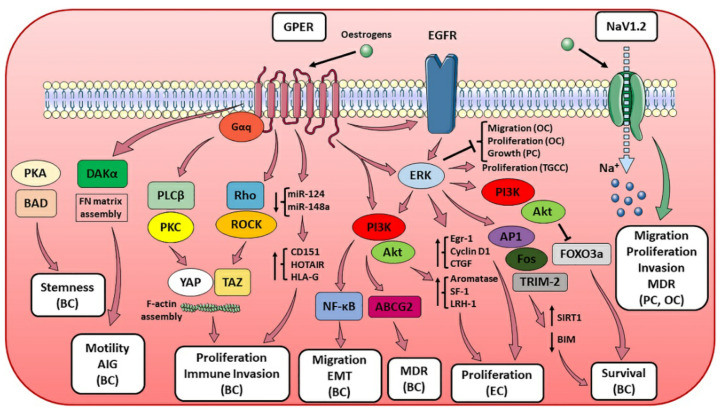
mER-associated pathways and their effect on hormone-sensitive cancer progression. The figure illustrates the molecular pathways correlated to the different mERs and their biological effects on multiple hormone-sensitive cancer types. When one or more tumor-related processes were reported only for a specific cancer type, the latter was made explicit in the figure and put in parentheses (e.g., breast cancer as BC, prostate cancer as PC, endometrial cancer as EC, ovarian cancer as OC and testicular germ cell cancer as TGCC). Other abbreviations: multi-drug resistance (MDR), anchorage-independent growth (AIG) (see text for details).

GPER tumorigenic properties have also been investigated in male cancers, namely, prostate and testicular cancers. Indeed, GPER activation led to the ERK1/2-mediated inhibition of prostate cancer cell growth [120], although its role and significance still need to be completely elucidated. Finally, GPER is also involved in regulating the proliferation of testicular germ cell cancer [121,122] and has been reported to be overexpressed in human seminoma (a testicular germ cell tumor subtype) [122]. Indeed, clinical and experimental studies support the hypothesis that estrogens, through GPER activity, contribute to the regulation of tumor testicular germ cells proliferation via ERK1/2 [121,122]. Noteworthy, two polymorphisms in the promoter region of GPER (rs3808350 and rs3808351) in seminomas are correlated to its overexpression and confer genetic susceptibility for testicular carcinogenesis [122]. Conversely, in Leydig cell tumor (a different testicular germ cell tumor subtype compared to seminoma), GPER activation is correlated with decreased proliferation, increased apoptosis [121] and perturbance of lipid metabolism and steroidogenesis via PI3K/Akt/mTOR pathway impairment [123], suggesting that, for testicular germ cell tumors, GPER-mediated effects on cell survival and proliferation depend on specific cell type (Figure 2).

#### 2.2.2. NaV1.2

Voltage-Gated Sodium Channels (VGSCs) are integral membrane glycoprotein complexes that, in response to membrane potential depolarization, undergo a conformational change, open a transmembrane pore and mediate a Na^+^ influx. VGSCs are composed of principal subunits alpha (VGSCα) and auxiliary subunits beta (VGSCβ), and, since they are functionally expressed in different tissues and cell types [124], VGSCs are involved in a variety of molecular events, including neurotransmission, nerve skeletal and cardiac muscle contraction, and secretion. In this regard, VGSCs play an important role in the development and progression of different cancers, since they have been correlated with different events of the carcinogenic process, including cell proliferation, migration, invasion, and Multi-Drug Resistance (MDR) [125,126]. NaV1.2 channel increased expression has been observed in different cancer types, including prostate [127] and ovarian cancer, where it is involved in regulating migration and invasion of highly metastatic cancer cells [128]. Interestingly, rapid estrogen actions on different ion channels’ functionality have been reported, and both endogenous and exogenous estrogens can bind NaV1.2, other VGSCs and other ion channels, to regulate their activity [129,130] (Figure 2).

### 2.3. Membrane Progesterone Receptors

In the same way for androgen and estrogen signaling, the progesterone receptor (PR), with its two isoforms PR-A and PR-B, is the most prominent and studied mediator of progestogenic effects, although other receptors localized at the plasma membrane level can participate in mediating progesterone (P4) and progesterone-like substances effects. These membrane progesterone receptors that mediate non-traditional progesterone actions are classified into two groups: the class II progestin and adipoQ receptor (PAQR) family (also called membrane Progesterone Receptors, mPRs) includes mPRα, mPRβ, mPRγ, mPRδ, mPRε encoded by PAQR7, PAQR8, PAQR5, PAQR6 and PAQR9 genes, respectively [131], while the b5-like haem/steroid-binding protein family (also called Membrane Associated Progesterone Receptors, MAPRs) comprises Progesterone Receptor Membrane Component 1 (PGRMC1), PGRMC2, GIG47 (previously known as Neuron-Derived Neurotrophic Factor, NENF, and neudesin) and neuferricin (alternatively termed Cytochrome B5 Domain Containing 2, CYB5D2) [132].

#### 2.3.1. mPRs

**Figure 3:**
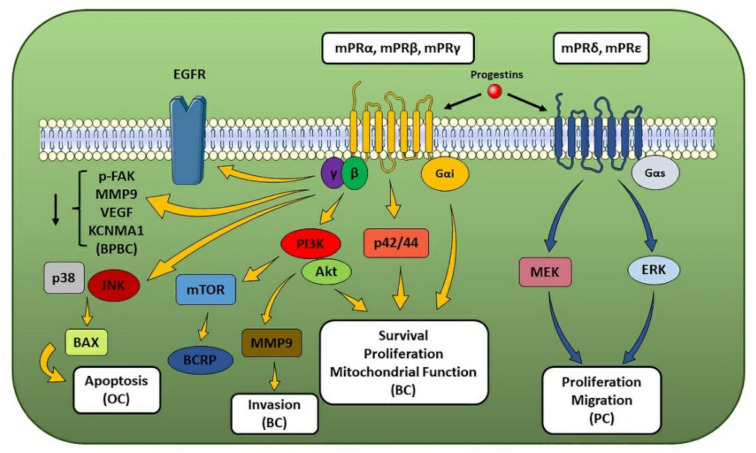
mPR-associated pathways and their effect on hormone-sensitive cancer progression. The figure illustrates the molecular pathways correlated to the different mPRs and their biological effects on multiple hormone-sensitive cancer types. When one or more tumor-related processes were reported only for a specific cancer type, the latter has been made explicit in the figure and put in parentheses (e.g., breast cancer as BC, prostate cancer as PC, ovarian cancer as OC, and basal phenotype breast cancer as BPBC) (see text for details).

The mPRs are 7-transmembrane protein receptors located in the cell plasma membrane that transduce via G-proteins, even though they do not share structural nor sequence homology to GPCRs and nuclear steroid receptors [131]. In mammals, these receptors show a differential expression in both reproductive (e.g., ovary, uterus, placenta, and testis) and non-reproductive (e.g., brain, kidney and intestinal) tissues [133] and display a high binding affinity for progestins. Although several members of the class II PAQR family are known, most literature data reported and focused on the functional characterization of mPRα, which is the most abundant mPR expressed in different human tissues [134], while information is still lacking for the other members. Several studies reported that mPRα, mPRβ and mPRγ are coupled and activate a Gαi, leading to a progestin-triggered down-regulation of adenylyl cyclase activity [131,134], while mPRδ and mPRε have been shown to signal through a Gαs [135]. In addition, after progestin activation, the βγ subunit has been reported to participate in signal transduction by upregulating PI3K/Akt, ERK1/2 and p38 MAPK molecular pathways [136]. Progestin functions correlated to the presence of mPRs and mPR-dependent signaling in mammalian cells have been suggested to mediate the development of breast and ovarian hormone-sensitive cancers [137,138] since P4 has been observed to inhibit the EMT process in breast cancer cells through mPRs-mediated PI3K and EGFR activation [139] and to transduce through mPRs in ovarian cancer cells [140]. Regarding breast cancer, mPRα and MMP9 expression and Akt phosphorylation have been reported to be higher in breast cancer tissue compared to non-cancerous one. A positive correlation between mPRα, HER2 and MMP9 expression and tumor size has been reported, while a negative correlation with ER and PR status has also been observed [141]. In addition, mPRα expression also showed a positive correlation with EGFR, HER2 and Ki67 expression pattern [142]. Indeed, P4-mediated mPRα activation has been found to modulate cell proliferation via PI3K molecular pathway [142]; to decrease apoptosis; to increase mitochondrial potential through Gαi, p42/44 MAPK and Akt signaling cascades [143]; and to upregulate the breast cancer resistance protein (BCRP)—an independent risk factor for breast cancer and possible marker for poor prognosis—through PI3K/Akt/mTOR pathway [144], supporting a role for mPRα in the development and progression of breast cancer through cell death inhibition and its role as a major prognostic marker of poor prognosis (Figure 3). Conversely, upon its P4-mediated activation, mPRα exerts inhibitory effects on cell proliferation and migration in triple-negative breast cancer [145] and in basal phenotype breast cancer via FAK dephosphorylation, MMP9, Vascular-Endothelial Growth Factor (VEGF), and Calcium-Activated Potassium Channel Subunit alpha-1 (KCNMA1, also known as BK channel alpha subunit or large conductance Ca^2+^-activated potassium channel, subfamily M, alpha member 1, KCa1.1) downregulation mechanisms [146]. Regarding ovarian cancer, expression profiles of mPRα and mPRβ suggested a potential role in the pathogenesis and development of ovarian tumors [147]. However, unlike in breast cancer cells, P4-triggered mPR activity has been shown to indirectly induce cAMP levels by enhancing β1,2-adrenergic receptor activation and to up-regulate apoptosis regulator BAX (also known as Bcl-2-like protein 4) through JNK1/2 and p38 MAPKs activation [148], indicating the existence of mPR-induced molecular pathway that, through pro-apoptotic MAPK signaling modules, can lead to ovarian cancer cell death and suggesting that P4-based hormone therapy could provide a suitable and effective strategy for the treatment of ovarian cancer [148] (Figure 3). In addition to breast and ovarian cancers, mPR have been associated also with other hormone-sensitive tumors like endometrial and prostate cancers. Indeed, analyses of samples of endometrial cancer patients not only revealed that mPRβ and mPRγ displayed a different subcellular localization in cancerous tissue compared to non-cancerous one but also suggest, based on expression data, these receptors as potential prognostic biomarker for endometrial cancer [149]. Regarding prostate cancer, mRNA expression analyses of samples from patients revealed that PAQR6 levels were upregulated compared to non-cancerous tissue and positively correlated with a lower survival rate. In addition, gene silencing data correlated mPRδ activity with MEK and ERK1/2 signaling pathway involved in proliferation and migration of tumor cells, suggesting its potential role in prostate cancer development and serving as potential prognosis biomarker [150] (Figure 3).

#### 2.3.2. MAPRs

MAPRs proteins, like mPRs, are membrane-bound receptors—evolutionarily conserved and distant homologues of the small haemoprotein Cytochrome B5 (CYB5)—able to bind P4 and activate rapid, non-genomic and PR-independent effects. MAPRs can interact with the superfamily of microsomal and mitochondrial haemoproteins Cytochrome P450 enzymes (CYP) and other different proteins to regulate a variety of molecular processes, including cell proliferation and migration, steroid homeostasis, and autophagy [132].

**Figure 4:**
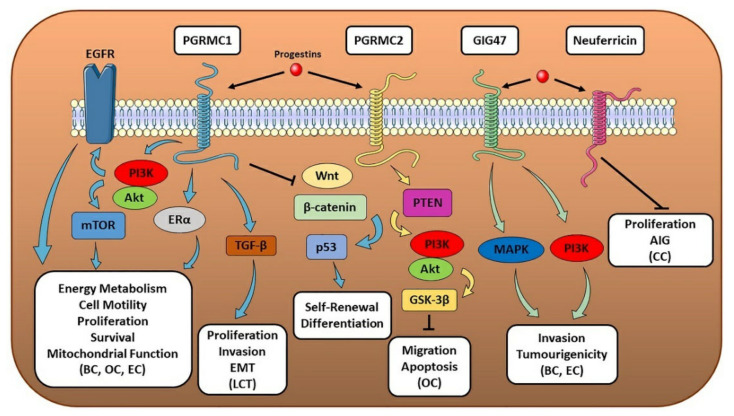
MAPR-associated pathways and their effect on hormone-sensitive cancer progression. The figure illustrates the molecular pathways correlated to the different MAPRs and their biological effects on multiple hormone-sensitive cancer types. When one or more tumor-related processes were reported only for a specific cancer type, the latter has been made explicit in the figure and put in parentheses (e.g., breast cancer as BC, endometrial cancer as EC, ovarian cancer as OC, Leydig cell tumor as LCT and cervical cancer as CC). Other abbreviations: anchorage-independent growth (AIG) (see text for details).

PGRMC1 features a single transmembrane domain and, besides progestins, binds several other ligands, including cholesterol, glucocorticoids, and other steroids [134]. It is localized, together with CYPs, in different subcellular compartments, including the plasma membrane, nucleus, endoplasmic reticulum and mitochondria [151]. Its expression is upregulated in different tumor types, and it is involved in promoting cancer progression and EMT [152,153]. This is in line with the reported role of PGRMC1 in promoting cell self-renewal and inhibiting differentiation by downregulating Wnt/β-catenin and p53 pathways [154], as well as upregulating energy metabolism, mitochondrial function, glycolysis, cell motility and tumor growth, through PI3K/Akt pathway [155]. Regarding hormone-sensitive cancers, PGRMC1 has been shown to regulate some breast cancer hallmarks [156]. Indeed, PGRMC1 can promote tumorigenesis [157], and its expression is associated with a malignant breast cancer phenotype [158]. Recently, independent studies showed a positive correlation between PGRMC1 expression levels and breast features associated with poor prognosis [159,160], further suggesting its role as tumor marker and strengthening PGRMC1 prognostic value for this malignancy. PGRMC1 can promote breast cancer development by increasing survival and growth of tumor cells [161] and inducing neovascularization in tumor tissue through VEGF activation [162]. These observations on its tumor-promoting role were confirmed by the reduced migration and metastasis of breast cancer cells after PGRMC1 knock-out [163] and by the suppression effect on PGRMC1 tumorigenicity exerted by miR-181a [164]. At molecular level, although conflicting data are reported in literature regarding PGRMC1-initiated effects in breast cancer cells [165], PGRMC1 activation enhances PI3K/Akt/mTOR and EGFR-mediated signaling pathways, resulting in the increased proliferation [166] and alteration of lipid metabolism [167] in ER+ and triple-negative breast tumors. This is in line with the reported association between PGRMC1 and ER levels [160], the observed PGRMC1-mediated ERα activation [167] and the increased breast cancer cell proliferation correlated with PGRMC1-ERα crosstalk recently reported [168]. However, PGRMC1’s role in tumor development and progression is not limited to breast cancer. Indeed, several studies reported its involvement also in other hormone-sensitive cancers, including testicular Leydig cell tumor proliferation and invasion through Transforming Growth Factor beta (TGF-β) alternative pathway [169], endometrial cancer [170] and ovarian cancer, in which PGRMC1 activation promotes cell viability, growth, proliferation, migration and survival through PI3K/Akt-mediated inhibition of apoptosis [171,172] (Figure 4).

PGRMC2 is a membrane-bound receptor structurally similar to PGRMC1, although it displays a different N-terminal transmembrane domain and is less functionally characterized [173]. Like PGRMC1, PGRMC2 is expressed in different tissues and with a similar subcellular localization. Although limited studies on its role in cancer development and progression are available, PGRMC2 expression, which has been shown to be post-transcriptionally regulated by miR-142-3p [174], is higher in breast cancer tissue compared to non-cancerous one, suggesting its potential contribution to the tumorigenesis of the breast [175] and serving as possible biomarker for breast cancer staging [176]. Moreover, PGRMC2 is highly expressed in ovarian cancer cells, and in vitro studies showed that its P4-mediated activation resulted in the inhibition of cell migration [177] and apoptosis through PI3K/Phosphatase and Tensin Homolog (PTEN)/Akt/GSK-3β axis [172] (Figure 4).

Although originally identified as a secreted protein with neurotrophic actions mediated via MAPK and PI3K pathways, Nuclear Magnetic Resonance (NMR) studies revealed that GIG47 shares a similar structure with PGRMC1 and 2 [178]. GIG47 increased expression has been reported in several cancer types, including uterine and breast cancer, in which it promotes cancer cell invasion and increased tumorigenicity through MAPK and PI3K pathways [178] (Figure 4). In addition to its receptor-correlated activity, an extracellularly secreted GIG47 has been shown to be involved in cancer cell immortalization and resistance to carcinogens [179].

A homology-based search of the same CYB5-like haem/steroid-binding domain of PGRMC1, PGRMC2 and GIG47 led to the discovery of Neuferricin that, like the other MAPRs, is expressed in a variety of different tissues [132]. Like GIG47, Neuferricin can be detected as both secreted haemoprotein and membrane-bound receptor, especially localized in the endoplasmic reticulum, where it can interact with CYP Reductase (POR) [180]. Its expression has been correlated with the inhibition of cell proliferation and anchorage-independent colony growth in vitro in cervical cancer cells [180] and has been suggested as a potential candidate tumor suppressor of cervical tumorigenesis [181]. However, correlations between Neuferricin expression or its activity and the development and progression of hormone-sensitive cancers have not been found yet.

## 3. Beyond mSRs Physiologic Role

Since the mSRs reported and dissected here show widespread expression in human tissues and can regulate the activation of well-known and pivotal signaling pathways, the exposure to exogenous hormone-active substances, e.g., EDCs, can cause their aberrant and non-physiologic activation. This can trigger different molecular mechanisms and initiate a variety of pleiotropic effects that, due to the uncertainty about time, duration, and concentration of exposure, may play a role in the development and progression of different diseases that rely on the activity of hormones and hormone-like compounds. Therefore, in a hormone-sensitive cancer context, mSR abnormal activation in different but adjacent tissues may shape the characteristics of the TME to drive and sustain the formation of the cancer mass [3]. On the other hand, this ability to affect not only cancer cells but also other cellular subpopulations within the TME, including immune cells, makes mSRs possible and interesting drug targets able to interfere not only with the biology of cancer cells but also with cancer-promoting and cancer-sustaining mechanisms activated by other adjacent cell types. In the following section, the role of different mSRs will be firstly dissected as possible TME shapers due to their role as EDCs receptors and, secondly, as potential drug targets for different hormone-sensitive cancers.

### 3.1. mSRs as EDCs Targets

In the classic definition of endocrine disruption, hormonally active agents are considered for their ability to interfere with the action of hormones and endocrine signaling primarily through nSRs. Noteworthy, EDCs have been shown to affect hormone signaling within the TME to promote a crosstalk between cancers cells, stromal cells, and immune cells and to influence the extracellular matrix remodeling in a pro-tumorigenic manner, finally contributing to cancer cell progression, invasion, and metastasis formation (reviewed in [3]). However, recent and increasing evidence suggests that also mSRs should be considered in the mechanism of endocrine disruption since EDCs have been shown to bind and modulate mSR-initiated downstream molecular processes.

Regarding mARs, in prostate cancer cells, a variety of pesticides, including prochloraz, vinclozolin and its metabolite M2 (3’,5’-dichloro-2-hydroxy-2-methylbut-3-enanilide), have been shown to exert antiandrogen actions through ZIP9, competing with testosterone and antagonising ERK-mediated pro-apoptotic gene BAX expression and reducing ZIP-mediated intracellular free Zn^2+^ influx [182]. However, potential adverse effects on human health as a consequence of the inhibition of ZIP9-dependent functions could include the attenuation of testosterone-activated pro-apoptotic and antitumorigenic effects in prostate cancer cells [9,12]. Moreover, in addition to environmental chemicals and pollutants, also plant-derived compounds have been shown to exert anti-androgenic and mAR-directed mechanism of endocrine disruption [32], although the roles of ZIP9, OXER1, and GPRC6A in mediating mechanisms of cancer progression activated by phyto-antiandrogens have still to be elucidated.

Among the different mERs, GPER is the most studied for its correlations with mechanisms of endocrine disruption [183,184]. In this regard, upon the binding of a variety of estrogen-active compounds—namely, diethylstilbestrol (DES), zearalenone (ZEA), bisphenols A, AF, B and S (BPA, BPAF, BPB and BPS), Tetrachlorobisphenol A (TCBPA) and Tetrabromobisphenol A (TBBPA) – GPER has been shown to enhance migration and proliferation of multiple breast cancer cell lines [185,186,187,188,189,190] by activating FAK, Src and ERK2 (via EGFR) to promote focal adhesion (FA) assembly [191]; to enhance cancer cell proliferation in testicular seminoma [192]; and, via PI3K/Akt/mTOR pathway, in Leydig cell tumor [193]; to induce, through a Receptor for Activated C Kinase 1 (RACK1)/PKC-dependent mechanism, an increased production of pro-inflammatory cytokines TNF-α and IL-8 in THP-1 cells, thus predisposing the cell to an increased response to pro-inflammatory stimuli [194,195,196]. These effects could be particularly relevant in shaping pivotal TME characteristics, since the misregulated and unhealthy Pro-tumor Inflammation (PTI), directly correlated with TNF-α and monocytes/macrophages action, has been shown to facilitate the oncogenic process by promoting tumor growth, survival and metastasis and to suppress the antitumoral immune response, leading to TME alterations [197]. In addition, 3-methylcholanthrene has been reported to activate EGFR/ERK/c-Fos transduction signaling through an Aryl hydrocarbon Receptor (AhR) and GPER-mediated mechanism in both breast cancer cells and Cancer-Associated Fibroblasts (CAFs). This activation led to the upregulation of cyclin D1 and CYP1B1 (an enzyme required for estrogen metabolism and oncogenic activation of environmental pollutants), which resulted in an increased tumor growth [198], further outlining GPER potential role in shaping the TME in a pro-tumorigenic manner.

On the other hand, literature data on mPR- and MAPR-related endocrine disruption mechanisms are still limited and uncertain. In this regard, in vitro analyses in MDA-MB-231 breast cancer cells showed that DES and its analogues can bind and activate mPRα, thus mimicking non-genomic progestins action [199]. In addition, the activation of PGRMC1 triggered by ethynylestradiol (EE) and other estrogen-like compounds has been shown to induce and enhance breast cancer cell proliferation both in vitro and in xenograft [200,201], in line with the previous observation that PGRMC1 can promote breast cancer cell proliferation through an E2-induced mechanism [202].

### 3.2. mSRs as Drug Targets

The ability to bind hormones and hormone-like substances and to activate non-genomic pathways with a pivotal role in cancer progression makes mSRs interesting and potential new drug targets for the development of structure-based compounds tailored to activate or antagonize one or more mSR, depending on the receptor, the activated pathways and the hormone-sensitive cancer considered. In addition, the expression of the same mSR in different but strictly connected tissues in the TME also offers the possibility to intervene not only on the tumorigenic process itself but also in cancer-sustaining mechanisms. Synthetic and natural compounds able to bind mSRs here presented—apart from TRPM8 and GPER, whose molecular modulators were recently and excellently reviewed [203,204]—are listed in Table 1.

Due to the relative novelty of mSRs as mediators of steroid signaling, studies on mSR-active compounds here presented were limited to the pre-clinical phase. Regarding ZIP9, both an antagonist and agonist compounds were analyzed for their ability to modulate ERK1/2, JNK and Bax pathways in both prostate and breast cancer cell lines with a final effect on apoptosis regulation. For OXER1, different antagonists were synthetized and screened both in vitro and in vivo, mostly for their ability to reduce inflammation-related pathways. In addition, several natural compounds were examined for their binding affinity for ZIP9, OXER1 and GPRC6A, although this analysis was limited to in silico data [227]. For mPRα and PGRMC1, an agonist and antagonist compounds have been reported, respectively. On the other hand, for CaV1.2, only antagonist compounds were described, even though cardiotoxicity or neurotoxocity were reported upon its blockade [228,229]. Finally, for GPRC6A, only an agonist has been reported, although an antagonist profile for its targeting could be more useful considering its inflammatory role regarding cytokine release [230]. Considering different and opposite tumor-dependent effects of the different mSRs, it will be important to develop targeted delivery systems to limit unwanted off-target effects.

## 4. Conclusions

Hormone-mediated rapid non-genomic effects, which cannot be attributed to the genomic action of nSRs, have raised attention on the potential involvement of mSRs in modulating specific molecular pathways pivotal for the physiologic hormone action. More recently, a possible contribution of mSRs to the development of hormone-linked diseases has been hypothesized, including hormone-related cancers. Because of their widespread expression in different human tissues, their role in promoting and driving not only the cancerous process itself but also other cancer-sustaining mechanisms in the surrounding TME Therefore, their ability to bind and exert the action of hormones and hormone-like substances (e.g., EDCs), also in a pathologic context, indicated, on one hand, their potential involvement in shaping the TME characteristic to support the tumor growth and, on the other, offered new insights into their potential as therapeutic targets. However, the limited knowledge on mSRs also as EDCs targets warrants further studies to dissect and elucidate their role in mediating non-physiologic mechanisms of endocrine disruption that can have an important impact on the development and progression of different hormone-linked diseases with a strong or hypothesized environmental component, including hormone-sensitive cancers. Moreover, the early-stage translational research on mSRs as novel drug targets requires additional studies for the development of new small molecules capable to specifically target these receptors and hinder or amplify their correlated action to counteract cancer development.

## Figures and Tables

**Table 1:** mSR-targeting compounds in pre-clinical studies and their correlated effects.

Compound	Receptor	Profile	Cell Line/Model	Pathway	Effect	Ref.
(−)-Epicatechin	ZIP9	agonist	PC3 (PC) MDA-MB-468 (BC)	ERK1/2, JNK and Bax	Proapoptotic action (increased Caspase-3 levels), increased cAMP and intracellular Zn^2+^ levels	[205]
(+)-Catechin	ZIP9	antagonist	PC3 MDA-MB-468	ERK 1/2, JNK and Bax		[205]
Bicalutamide	ZIP9	antagonist	93RS2 (non -cancerous testicular cell line)	ERK1/2, CREB and ATF-1	Reduced claudin-5 and zonula occludens-1 (ZO-1) expression	[206]
Nandrolone	OXER1	antagonist	MCF-7, MDA-MB-231 (BC)	PI3K/Akt/NF-κB and RACK1	Reduced proliferation and migration	[35]
5-oxo-EPE	OXER1	agonist	In vitro assay		Increased β-Arrestin recruitment	[207]
S-230	OXER1	antagonist	In vivo (monkeys), human neutrophils	Gβγ-mediated signaling	Reduced Gβγ-mediated Ca^2+^ mobilization	[208,209]
S-Y048	OXER1	antagonist	In vivo (monkeys), human neutrophils and human eosinophils	Gβγ-mediated signaling	Reduced Gβγ-mediated Ca^2+^ mobilization, actin polymerization and eosinophil infiltration	[210]
S-C025	OXER1	antagonist	In vivo (monkeys), human neutrophils	Gβγ-mediated signaling	Reduced Gβγ-mediated Ca^2+^ mobilization and eosinophil activation	[211]
264	OXER1	antagonist	In vivo (monkeys, rats), monkey eosinophils and monkey neutrophils	Gβγ-mediated signaling	Reduced Gβγ-mediated Ca^2+^ mobilization, actin polymerization and chemotaxis in granulocytes	[212]
DJ-V-159	GPRC6A	agonist	HEK-293 (human embryonic kidney), MIN-6 (mouse pancreatic β-cell) and in vivo (mice)	Gαs-dependent signaling, ERK1/2	Increased cAMP levels, insulin secretion and decreased serum glucose (in vivo, mouse)	[213]
Diltiazem	CaV1.2	antagonist	In vivo (mice)	CaV1.2-PKC	Inhibition of Ca^2+^ influx, PLCδ1	[214]
Lercanidipine	CaV1.2	antagonist	Healthy and pediatric acute myeloid leukemia (AML) mesenchymal stromal cells (MSCs)		Inhibition of Ca^2+^ influx	[215]
Ketamine	CaV1.2	antagonist	In vivo (mice), *Xenopus laevis* oocytes (ex vivo)		Inhibition of CaV1.2 expression; Ca^2+^ influx; and vascular smooth muscle contraction	[216]
Ritanserin	CaV1.2	antagonist	Rat vascular myocytes (ex vivo)		Inhibition of Ca^2+^ influx; in vitro vasodilation; and vascular smooth muscle relaxation	[217]
(R)-Roscovitine	CaV1.2	antagonist	HEK-293		Slows activation and enhances inactivation	[218]
Metergoline	NaV1.2	antagonist	*Xenopus laevis* oocytes (ex vivo)		Inhibition of Na^+^ influx	[219]
Ranolazine	NaV1.2	antagonist	CHO		Inhibition of Na^+^ influx	[220]
2,4(5)-diarylimidazoles	NaV1.2	antagonist	In vitro assay		Inhibition of Na^+^ influx	[221]
Org OD 02-0	mPRα	agonist	A549, PC-9 (human lung adenocarcinoma), HBE (human bronchial epithelial) and MCF-7	PKA/CREB and PKA/β-catenin	Inhibition of cell growth and tumor growth (in vivo)	[222]
Ganaxolone	mPRδ	agonist	GT1-7 (rat hypothalamic cells), H19-7 (rat hippocampal neuronal cells)	Gαs-dependent signaling	Reduction of apoptosis and cell death	[223]
AG-205	PGRMC1	antagonist	CHO-K1, HeLa, COS-7, and H4 glioma Cells		Increased endosome formation	[224]
			PaCa-2 cells (pancreatic cancer)	RACK1, alpha-Actinin-1	Reduced PGRMC1 interactions with the actin cytoskeleton	[225]
			Human granulosa/luteal cell	B-cell lymphoma 2 (BCL2) pathway	Increased PGRMC1 monomeric form, increased proapoptotic Harakiri (Hrk) expression	[226]
